# Ephaptic Coupling Is a Mechanism of Conduction Reserve During Reduced Gap Junction Coupling

**DOI:** 10.3389/fphys.2022.848019

**Published:** 2022-05-05

**Authors:** Joyce Lin, Anand Abraham, Sharon A. George, Amara Greer-Short, Grace A. Blair, Angel Moreno, Bridget R. Alber, Matthew W. Kay, Steven Poelzing

**Affiliations:** ^1^ Department of Mathematics, California Polytechnic State University, San Luis Obispo, CA, United States; ^2^ Virginia Tech Carilion School of Medicine, Roanoke, VA, United States; ^3^ Fralin Biomedical Research Institute at Virginia Tech Carilion School of Medicine, Roanoke, VA, United States; ^4^ Biomedical Engineering and Mechanics, Virginia Tech, Blacksburg, VA, United States; ^5^ Translational Biology, Medicine and Health, Virginia Tech, Roanoke, VA, United States; ^6^ Department of Biomedical Engineering, The George Washington University, Washington, DC, United States

**Keywords:** cellular coupling, propagation, gap junction remodeling, myocardium, simulation

## Abstract

Many cardiac pathologies are associated with reduced gap junction (GJ) coupling, an important modulator of cardiac conduction velocity (CV). However, the relationship between phenotype and functional expression of the connexin GJ family of proteins is controversial. For example, a 50% reduction of GJ coupling has been shown to have little impact on myocardial CV due to a concept known as conduction reserve. This can be explained by the ephaptic coupling (EpC) theory whereby conduction is maintained by a combination of low GJ coupling and increased electrical fields generated in the sodium channel rich clefts between neighboring myocytes. At the same time, low GJ coupling may also increase intracellular charge accumulation within myocytes, resulting in a faster transmembrane potential rate of change during depolarization (*dV/dt_max*) that maintains macroscopic conduction. To provide insight into the prevalence of these two phenomena during pathological conditions, we investigated the relationship between EpC and charge accumulation within the setting of GJ remodeling using multicellular simulations and companion perfused mouse heart experiments. Conduction along a fiber of myocardial cells was simulated for a range of GJ conditions. The model incorporated intercellular variations, including GJ coupling conductance and distribution, cell-to-cell separation in the intercalated disc (perinexal width—W_P_), and variations in sodium channel distribution. Perfused heart studies having conditions analogous to those of the simulations were performed using wild type mice and mice heterozygous null for the connexin gene Gja1. With insight from simulations, the relative contributions of EpC and charge accumulation on action potential parameters and conduction velocities were analyzed. Both simulation and experimental results support a common conclusion that low GJ coupling decreases and narrowing W_P_ increases the rate of the AP upstroke when sodium channels are densely expressed at the ends of myocytes, indicating that conduction reserve is more dependent on EpC than charge accumulation during GJ uncoupling.

## Introduction

Nearly two-thirds of sudden cardiac deaths in the United States are caused by arrhythmias (Srinivasan and Schilling, 2018) that have multiple mechanisms. Reentrant arrhythmias occur under conditions where an excitation wavefront fails to propagate into temporarily inexcitable tissue (unidirectional conduction block) and instead propagates into nearby excitable tissue (Mines, 1913). Given sufficient time and an appropriate path length, a wavefront could reenter the previously inexcitable tissue and excite it, creating a reentrant circuit of electrical excitation that could persist as a tachycardia. Slowed conduction is one such mechanism for increasing the time between unidirectional block and reentry into the site of block after the tissue regains excitability. Thus, arrhythmia initiation is associated with two mechanisms tied to local excitation: tissue excitability and electrical wavefront conduction velocity (CV).

Excitability and CV are related by their biophysical dependence on similar membrane-bound proteins, electrochemical forces, and cellular structures, but these parameters are not linearly correlated. The relationship of gap junction (GJ) uncoupling to tissue excitability and CV underscores the complex interaction between these parameters. Specifically, CV does not always dramatically change during GJ uncoupling (Shaw and Rudy, 1997b), an outcome that has been explained by the theory of conduction reserve (Stein et al., 2006).

One mechanism of conduction reserve predicts that cellular uncoupling reduces electronic load while concurrently reducing the propensity for conduction failure, because charge accumulates within the intracellular space of individual cardiomyocytes (Spach et al., 1988; Spach et al., 1992; Spach and Heidlage, 1995; Shaw and Rudy, 1997a). As a result, the elevated intracellular charge would result in the faster movement of action potentials (AP) through cells yet slower movement between cells due to cellular uncoupling (Diaz et al., 1983). The net effect would be that CV would not substantively change for modest levels of GJ uncoupling. Evidence of charge accumulation in cardiomyocytes might be a sharper AP upstroke, as the maximum slope of transmembrane potential (*dV/dt_max*) is often used as a measure of cellular excitability. Conduction reserve theory was developed in part to explain the observation that *dV/dt_max* is higher when the AP wavefront propagates along the short (transverse) axis relative to the long (longitudinal) axis of cardiomyocytes since intercellular resistance through GJ is higher transverse to myocytes (Spach et al., 1981).

Computationally, increased *dV/dt_max* has been reported by some (Joyner, 1982; Spach and Kootsey, 1985; Spach et al., 1987; Spach et al., 1988; Shaw and Rudy, 1997b) but not all models of conduction in response to GJ uncoupling (Thomas et al., 2003; Boyle et al., 2019). The experimental evidence is not entirely consistent either. For example, we and others have reported that *dV/dt_max* increases, does not change, or decreases as conduction slows secondary to GJ uncoupling (Thomas et al., 2003; Delmar et al., 1987; Jalife et al., 1989; Rohr et al., 1998; Ozaki et al., 1989; Degroot et al., 2003; Poelzing and Rosenbaum, 2004; Dhillon et al., 2013; Entz et al., 2016; O'Shea et al., 2019).

The relationship between slowed CV and conduction failure is particularly important during ischemia, an event which is associated with collapse of the extracellular space (Tranum-Jensen et al., 1981; Kléber et al., 1987; Beardslee et al., 2000), followed by GJ uncoupling (Beardslee et al., 2000). Recent evidence suggests that bulk extracellular volume collapse occurs in parallel with extracellular fluid accumulation at the intercalated discs between cardiomyocytes, specifically within an extracellular nanodomain adjacent to the GJ called the perinexus (George et al., 2019; Hoeker et al., 2020). The perinexus has been proposed to facilitate another form of direct electrical communication between cardiomyocytes called ephaptic coupling (EpC), which is brought about by enrichment of the voltage gated sodium channel Nav1.5 (Veeraraghavan et al., 2015) and the inward rectifier potassium channel Kir2.1 (Veeraraghavan et al., 2016) within the perinexus. EpC occurs by a concurrent change in extracellular potential and ion concentrations that are important for determining the transmembrane potential and ionic reversal potentials of the ion channels in the apposing membrane (Rhett et al., 2011; Veeraraghavan et al., 2015). As a result, EpC is another mechanism that sustains conduction during altered excitability. While the relationship between *dV/dt_max* and GJ uncoupling is well-researched, previous studies that evaluated this relationship did not include EpC. This is important because GJ coupling and EpC modulate conduction simultaneously and in a compensatory manner. Therefore, the purpose of this study was to determine whether intracellular charge accumulation or ephaptic coupling is a primary mechanism of conduction reserve during reduced GJ coupling. Specifically, we investigated how *dV/dt_max* and AP *rise time* are altered in response to functional GJ reduction. Both simulation and experimental results support a common conclusion that enhanced EpC, rather than charge accumulation, increases AP rate of rise, which preserves CV during GJ uncoupling.

## Methods

The relationship between EpC and the rate of membrane depolarization within the setting of altered GJ coupling was investigated using multicellular simulations and perfused mouse heart experiments. Results were analyzed to assess the relative contributions of EpC and intracellular charge accumulation on action potential parameters and CVs.

### Simulations

**FIGURE 1:**
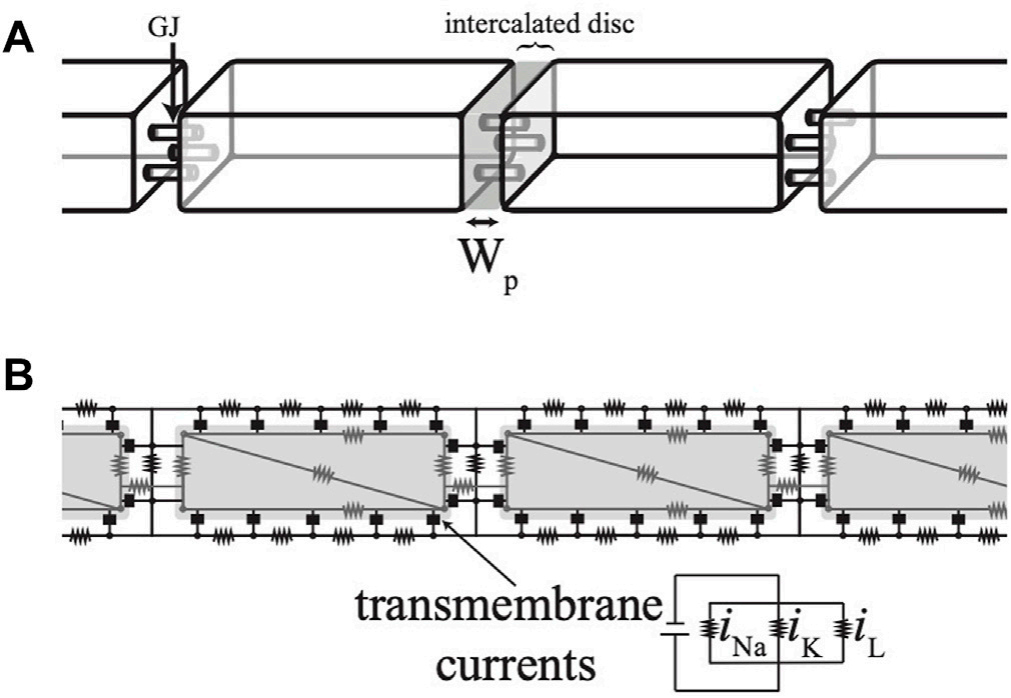
**(A)** Simulations were conducted on a strand of cells modeled as rectangular prisms. **(B)** A top-down cross-section of the cells shows the numerical scheme as an electrical circuit.

Action potential propagation along a one-dimensional fiber of cardiomyocytes was simulated using a membrane model of intermediate complexity, as previously described (Veeraraghavan et al., 2015; Veeraraghavan et al., 2016; King et al., 2021). Fifteen cells, modeled as rectangular prisms, were connected end-to-end via GJs (Figure 1A). These GJs were placed on the cellular membrane, only in the intercalated disc. A steady-state Hodgkin and Huxley model was used for the ionic currents, representing sodium, potassium, and leakage currents. The exact formulation for this ionic current equation is in Lin & Keener (2010), Appendix (Lin and Keener, 2010). Sodium and potassium ion channels were either spread uniformly over the entire membrane of the cell or primarily located at the intercalated discs at the ends of each cell. Physiological parameters were chosen using values from previously studied models in the literature. Myocytes were 0.01 cm × 0.00167 cm × 0.00167 cm in size (Hand and Griffith, 2010) and had an intracellular conductance of 6.7 mS/cm (Shaw and Rudy, 1997b; Lin and Keener, 2014) and an extracellular conductance of 14.8 mS/cm (Neu and Krassowska, 1993; Veeraraghavan et al., 2015). GJ coupling had a nominal value of 600 mS/cm^2^ (Rohr et al., 1998; Lin and Keener, 2014). Extracellular width along the lateral membrane of the cells was held constant at 1 × 10^−5^ cm while cell-to-cell separation, the width of the intercalated disc (W_P_), varied from a nominal value of 1.5 × 10^−6^ cm (Lin and Keener, 2010; Lin and Keener, 2014).

The interior of each cell was discretized using finite elements with four nodes. The extracellular space was discretized along two dimensions with the assumption that the extracellular potential along the shortest dimension was uniform (Lin and Keener, 2010; Lin and Keener, 2014). The nodes and connections are diagramed as an electrical circuit, as shown in Figure 1B. Current was injected into one end of the fiber to initiate a propagating action potential, after which the current was removed and no-flux boundary conditions were imposed. The time of membrane depolarization was determined using a transmembrane potential threshold. Conduction velocity along the fiber was computed using the times of depolarization at a point 1/3 down the length of the fiber and at another point 2/3 down the length of the fiber.

### Experiments

Perfused heart studies were performed using wild-type (WT) C57BL/6 mice and C57BL/6 transgenic mice heterozygous (HZ) for connexin43 (Cx43) null mutations that resulted in a 50% reduction of Cx43 expression compared to WT (Guerrero et al., 1997). Cx43 HZ mice were genotyped by Transnetyx (Cordova, TN) to confirm the Cx43 null mutation. All animal protocols conformed to the Guide for the Care and Use of Laboratory Animals published by the US National Institutes of Health (NIH Publication No. 85−23, revised 1996) and was approved by the Institutional Animal Care and Use Committee of the Virginia Tech Carilion Research Institute (George et al., 2015).

#### Langendorff Perfusion

WT and Cx43 HZ mice, 10–30 weeks of age, were anesthetized by a lethal intraperitoneal injection of sodium pentobarbital (325 mg/kg). Hearts were quickly excised after confirming a surgical plane of anesthesia via the lack of pedal reflex. The aorta was cannulated and hearts were retrograde perfused at a constant pressure of 65 mmHg that provided coronary flow rates between 1 and 1.5 ml/min, as previously described (Morley et al., 1999; Eloff et al., 2001). Perfusate was maintained at a pH of 7.4, 37°C, and oxygenated with 95/5 gas. The superfusion bath was maintained at 37°C. During specific experiments, perfusate was switched between the two solutions presented in Table 1, where one had a Ca^2+^ concentration of 1.8 mM and the other had a Ca^2+^ concentration of 3.4 mM. Other perfusate compounds were modulated slightly to maintain the same osmolarity between the two solutions, which was confirmed using a Precision Systems Micro Osmometer. Hearts were perfused with the two solutions in random order to minimize the effects of perfusion order and time.

**TABLE 1:** Composition of the two perfusate solutions used to modulate perinexal width (W_P_) during the perfused mouse heart experiments. Osmolarity values presented as mean ± standard deviation.

Tyrode composition (mM)	1	2
NaCl	130	126.2
NaHCO_3_	24	29
NaH_2_PO_4_	1.2	
Total [Na^+^]	155.2	155.2
KCl	4	3
KH_2_PO_4_		1
Total [K^+^]	4	4
MgCl_2_	1	
MgSO_4_		1
Glucose	5.6	10
CaCl2	1.8	3.4
BDM	15	10
Osmolarity (mOsm)	318.8 ± 0.9	323.6 ± 0.8

#### Transmission Electron Microscopy

Cardiac perinexal width was measured using transmission electron microscopy (TEM) in a separate set of experiments. After the hearts of WT and Cx43 HZ mice were perfused for 1 h, a cube of right ventricular tissue (1 mm^3^) was harvested, fixed in 2.5% glutaraldehyde overnight at 4°C, and then transferred to phosphate buffered saline at 4°C. The tissue was processed as previously described (Veeraraghavan et al., 2015), initially in 1% osmium tetroxide (0s04) and 1.5% potassium ferricyanide followed by rinsing with H_2_O. Samples were transferred to ethanol at increasing concentrations (70, 95, and 100%) for 15 min at each concentration and then transferred to a 1:1 solution of 100% ethanol and acetonitrile for 10 min. Samples were then transferred to only acetonitrile for two 10 min intervals and then embedded in PolyBed 812 at increasing concentrations with acetonitrile on a rotator. The samples were left in vacuum for ∼3 h and then left in PolyBed 812 overnight and transferred to flat molds and incubated at 60°C for 2 days. The blocks were sectioned using a microtome onto copper grids and stained with uranyl acetate (aq) for 40 min followed by Hanaichi Pb stain. Images of the GJs and the perinexus were acquired at 150,000x using a transmission electron microscope (JEOL JEM 1400). The perinexal width (W_P_) in those images was measured using ImageJ.

#### Glass Microelectrode Recordings

The transmembrane potential of left ventricular myocytes was measured *in situ* for a subset of WT and Cx43 HZ mouse hearts perfused with a solution having 1.8 mM Ca^2+^ and paced at a constant cycle length of 150 msec. Transmembrane potential was measured using our previously described modified glass microelectrodes that resembled a bee’s stinger (Barbic et al., 2017). Briefly, filamented glass micropipettes were heat-pulled using a conventional micropipette puller (Model PP-830, Narishige) then back-filled with 3 M KCl solution. The tip was broken off, fitted with a 25 μm diameter chloridized silver wire, and the back of the tip was sealed with a small bead of melted wax. The other end of the wire was connected to the headstage of an intracellular pre-amplifier (Duo 773 Electrometer, World Precision Instruments). A reference Ag/AgCl pellet was submersed in the bath superfusate and connected to the ground terminal of the headstage. Transmembrane potential signals were then continuously acquired from the 10X output channel of the intracellular pre-amplifier using a PowerLab (ADInstruments) data acquisition unit. The glass microelectrode was very slowly advanced to impale the medial-lateral epicardial surface of the left ventricle, placed transverse to the axis of propagation from the stimulating electrode, until steady state APs were observed. Transmembrane potential signals were then acquired and monitored in real time using LabChart (ADInstruments) software for the duration of the experiment. The average time to peak of the action potential (time from the end of phase 4 to the peak of phase 0 depolarization) of 50 epicardially paced action potentials (replicates) for each heart was obtained, and the average of the replicate average was statistically analyzed. All data, and the mean and standard deviation of the average of the replicates are presented.

#### Optical Mapping

Perfused hearts were electromechanically uncoupled using 2,3-butanedione monoxime (BDM) at a concentration of either 10 or 15 mM (Table 1). Hearts were stained with the voltage-sensitive dye di-4-ANEPPS for approximately 5 min at a concentration of 4 μM. Any residual contractile motion was minimized by applying slight pressure to the back of the heart to stabilize the anterior surface against the front glass of the superfusion bath. To excite the dye, the anterior surface was illuminated with bandpass filtered light (510 nm, Brightline Fluorescence Filter) emitted from a halogen lamp (MHAB-150 W, Moritex Corporation). Light emitted from the heart was longpass filtered [610-nm, 610FG01- 50 (T257), Andover Corporation] before it was imaged using a MiCam Ultima CMOS L-camera (100 × 100 pixels) at a rate of 1,000 frames/sec. The optical mapping field of view was 1 cm^2^ with an interpixel resolution of 0.1 mm. Hearts were paced using a unipolar silver wire positioned at the center of the anterior surface. A reference electrode was placed in the bath behind the heart. Hearts were stimulated at −1 V for 1 ms at a basic cycle length of 150 ms. Activation times were assigned to the maximum rate of rise of an AP, as previously reported (Veeraraghavan et al., 2012).

#### Conduction Velocities

CV vector fields for the optical mapping data were calculated using the Bayly et al. algorithm (Bayly et al., 1998), where a polynomial surface was fitted to local activation times to provide a CV vector at each pixel. Transverse conduction velocity was analyzed in this study for two reasons. First, our prior experience suggests that epicardial optical mapping estimates transverse conduction velocity with a higher accuracy because of the increased number of vectors (replicates) obtained in the transverse relative to longitudinal direction that meets the data inclusion criteria. Second, increased *dV/dt_max* in the transverse direction of propagation is a foundational observation relating *dV/dt_max* to GJ uncoupling (Spach et al., 1981).

#### Analysis of Conduction

A custom written program in Matlab was used to measure rise time by first identifying the AP upstroke and measuring the time between 10 and 90% of optical AP amplitude. For each experiment, rise time was estimated from a minimum of 10 sites along the transverse axes of propagation and then averaged (Entz et al., 2016).

## Results

### Simulations

**FIGURE 2:**
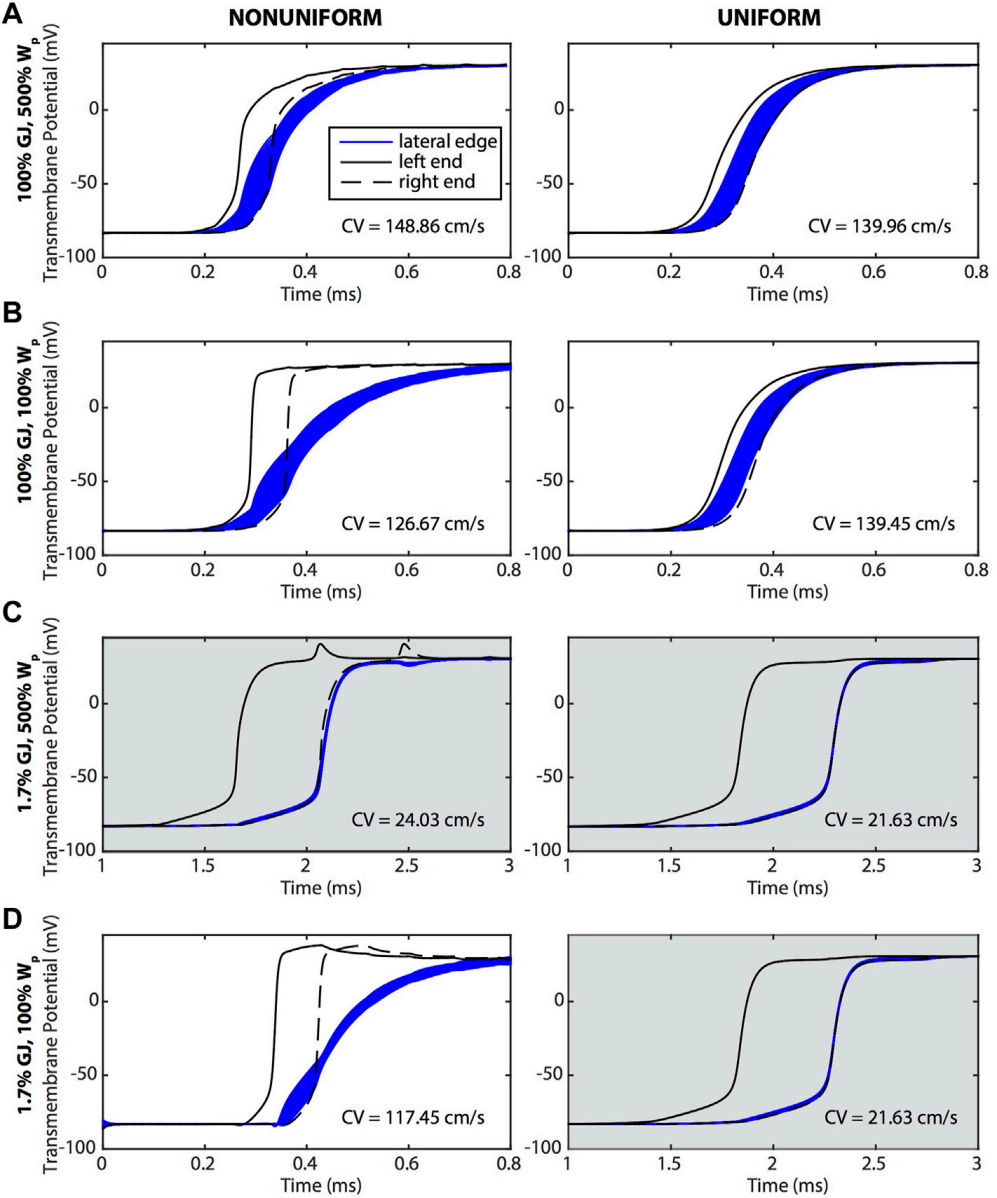
Transmembrane potentials along the ends (black) and lateral edges (blue) of a cell undergoing an AP that is traveling from left to right. The left column (nonuniform) shows cells with sodium and potassium ion channels preferentially distributed on the ends of the cells, and the right column (uniform) shows cells in which ion channels were evenly distributed over the surface of the cell. **(A)** The widest W_P_ case was compared against tissue where **(B)** W_p_ was nominal, **(C)** GJ coupling was reduced and W_p_ was wide, and **(D)** W_p_ was nominal and GJ coupling reduced. In general, APs traveling along the length of the cell were responding to the depolarization at the ends of the cell. The CVs for each case are reported on the graphs. Gray shading indicates panels having a longer time scale due to slower conduction velocities.

The perinexus facilitates direct electrical communication between cardiomyocytes. To enhance or reduce this effect, we varied GJ coupling and the width of the perinexus (W_P_). Action potential propagation along a single strand of cardiomyocytes was simulated with GJ coupling ranging from low (10 mS/cm^2^) to nominal (600 mS/cm^2^) and W_P_ ranging from nominal (1.5 × 10^−6^ cm) to high (7.5 × 10^−6^ cm). Transmembrane potentials at the ends (intercalated discs) and along the edges (lateral sides) of a single cell in the middle of the strand are plotted in Figure 2 for an AP traveling from left to right. Potentials for cells having sodium and potassium ion channels predominantly (90%) located at the ends of the cells (nonuniform coupling with enhanced ephaptic coupling) are shown in the left column of Figure 2. Potentials for cells with ion channels uniformly distributed across the cell surface (labeled uniform with reduced ephaptic coupling) are shown in the right column of Figure 2.

A preferential distribution of sodium and potassium channels at the ends of the cells (non-uniform coupling distribution) resulted in a dramatic delay of membrane depolarization along the edges compared to a uniform coupling distribution (Figure 2). In comparing the left and right columns of Figure 2, cells with ion channels primarily at the ends depolarized quickly at the ends and more slowly along the edges of the cell. This is seen in the graphs where the bulk of the edge membrane potentials (blue curves) are still rising after the rapid upstroke of the potentials at the ends (black curves). These results demonstrate that AP propagation from one end of the cell to the other end was dictated primarily by depolarization at the ends of the cells, with the edge membrane depolarizing later, recapitulating a form of saltatory conduction. In contrast, for cells having uniformly distributed ion channels, AP propagation proceeded sequentially with the upstream end of the cell depolarizing first, followed by the edges, and ending with depolarization at the other end of the cell.

Reducing W_P_ when GJ coupling was either normal or low had a more pronounced effect on membrane depolarization for a non-uniform compared to uniform distribution of ion channels. For example, reducing W_P_ within the setting of normal GJ coupling (Figures 2A,B) resulted in a larger surface area of the membrane edges having a slower depolarization phase than that of the ends. Reducing W_P_ also prolonged the depolarization phase along the edges of the membrane and shortened it at the ends. The effect of reducing W_P_ on membrane depolarization was even more pronounced within the setting of low GJ coupling (Figures 2C,D). With low GJ coupling and a non-uniform distribution of ion channels, reducing W_P_ dramatically slowed membrane edge depolarizations, with full depolarization occurring much later than at the ends while, interestingly, CV increased dramatically. Regardless of GJ coupling, when ion channels were uniformly distributed, membrane depolarization and CV were insensitive to reductions in W_P_.

A reduction of GJ coupling when W_P_ was large, regardless of ion channel distribution (Figures 2A,C), resulted in a dramatic reduction in CV and synchronized depolarization of the edges of the cell with the downstream end of the cell. A reduction of GJ coupling when W_P_ was nominal (Figures 2B,D, right) also resulted in a dramatic reduction of CV when ion channels were uniformly distributed. However, CV was maintained for low GJ coupling and nominal W_P_ when ion channels were non-uniformly distributed (Figure 2D, left).

**FIGURE 3:**
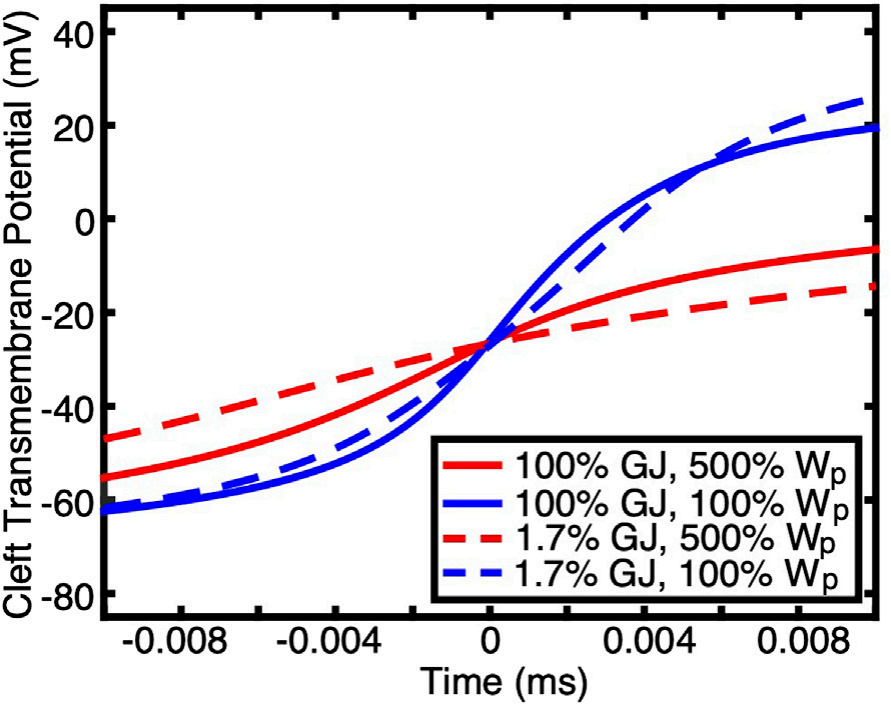
Transmembrane potentials during the depolarization phase of APs at one end of a cell (the membrane at the intercalated disc) are shown for simulations having non-uniform ion channel distribution with different GJ coupling and W_p_. Time was shifted such that the midpoint of the upstroke occurred at 0 ms. W_p_ had a greater effect on the rate of depolarization than GJ coupling.

The physiological relevance of CV being maintained during a dramatic reduction in GJ coupling if ion channels are non-uniformly distributed is supported by the fact that the cardiomyocytes of mature myocardium do indeed have potassium and sodium channels primarily distributed at the ends of the cells (Milstein et al., 2012; George et al., 2015; Veeraraghavan et al., 2015). Our simulation results demonstrate that without both this preferential distribution and small perinexal clefts, conduction would not be preserved during GJ uncoupling. Furthermore, W_P_ had a much greater effect than GJ coupling on the rate of transmembrane depolarization during phase zero of the AP when ion channels were preferentially located at the ends of the cells (Figure 3). The maximum rate of depolarization for large perinexal width was less than that of nominal width regardless of GJ coupling (Figure 3, red versus blue). For both values of W_P_, there was a more modest reduction in the rate of AP depolarization during low GJ coupling (Figure 3, solid versus dashed lines). The finding that a dramatic reduction in GJ coupling modestly decreases the rate of depolarization indicates that, for the conditions that were simulated, charge accumulation may not be the primary mechanism for conduction preservation during GJ uncoupling.

**FIGURE 4:**
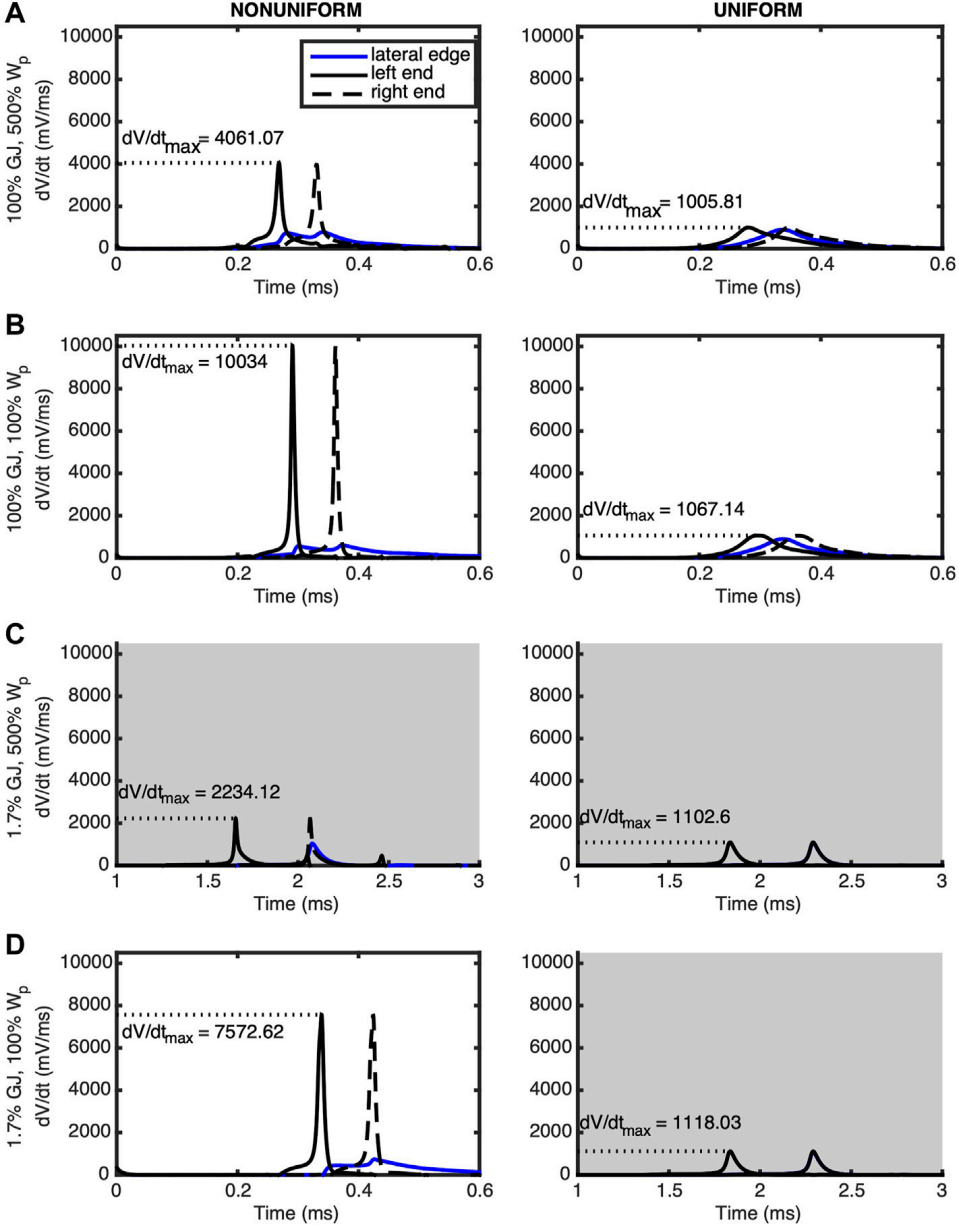
The derivative of the transmembrane potential is plotted for the left (solid black) and right (dashed black) ends as well as the lateral midpoint (blue) of a cell undergoing an AP traveling from left to right. Panels are arranged in the same positions as those of Figure 1. Left column (nonuniform) shows cells with sodium and potassium ion channels preferentially distributed at the ends of the cells. Right column (uniform) shows cells with ion channels evenly distributed over the surface of the cell. **(A)** The wide W_P_ case was compared against tissue where **(B)** W_p_ was nominal, **(C)** GJ coupling was reduced and W_p_ was wide, and **(D)** W_p_ was nominal and GJ coupling reduced. In the nonuniform cases, dV/dt_max is more sensitive to W_p_ compared to GJ coupling. Gray shading indicates panels having a longer time scale due to slower conduction velocities.

Detailed insight into the modulation of the rate of membrane depolarization during phase zero of the AP is provided in the plots of membrane potential derivative (*dV/dt*) shown in Figure 4. For nonuniform ion channel distribution and normal (100%) GJ coupling, the following was observed at the middle of the edge membrane: 1) depolarization occurred *after* each of the ends depolarized, when the *dV/dt* for the ends was decreasing, and 2) edge membrane *dV/dt* had two distinct peaks, indicating that the edge membrane depolarized in response to the depolarization of *each* end of the cell (Figures 4A,B). For nonuniform ion channel distribution and reduced (1.7%) GJ coupling, the middle of the edge membrane depolarized primarily in response to, and after, only the right (downstream) end of the cell (Figures 4C,D). Furthermore, with nonuniform ion channel distribution, maximum *dV/dt (dV/dt_max)* increased when W_P_ was reduced while GJ coupling remained either normal or low (Figures 4A–D). *dV/dt_max* decreased at the ends of the myocyte to a lesser extent during GJ uncoupling regardless of W_P_ (Figures 4A–D), indicating that for nonuniform ion channel distribution *dV/dt_max* was most sensitive to W_P_. Careful examination of the left column of Figure 4, rows a & c and rows b & d, also reveal that GJ uncoupling increased *dV/dt_max* modestly in the remaining sodium channels along the lateral membrane (blue lines).

When ion channels were uniformly distributed (Figure 4, right column) and GJ coupling was normal, regardless of W_P_, the middle edge membrane depolarized after the left end and before the right end, as expected (Figures 4A,B). The insignificance of reducing W_P_ for uniform ion channel distribution was further revealed when GJ coupling was lowered, whereby the middle of the edge membrane depolarized at the same time as the right end of the cell, regardless of W_P_. Finally, for uniform ion channel distribution, maximum *dV/dt_max* did not change significantly for any simulated combination of W_P_ and GJ coupling.

### Experiments

**FIGURE 5:**
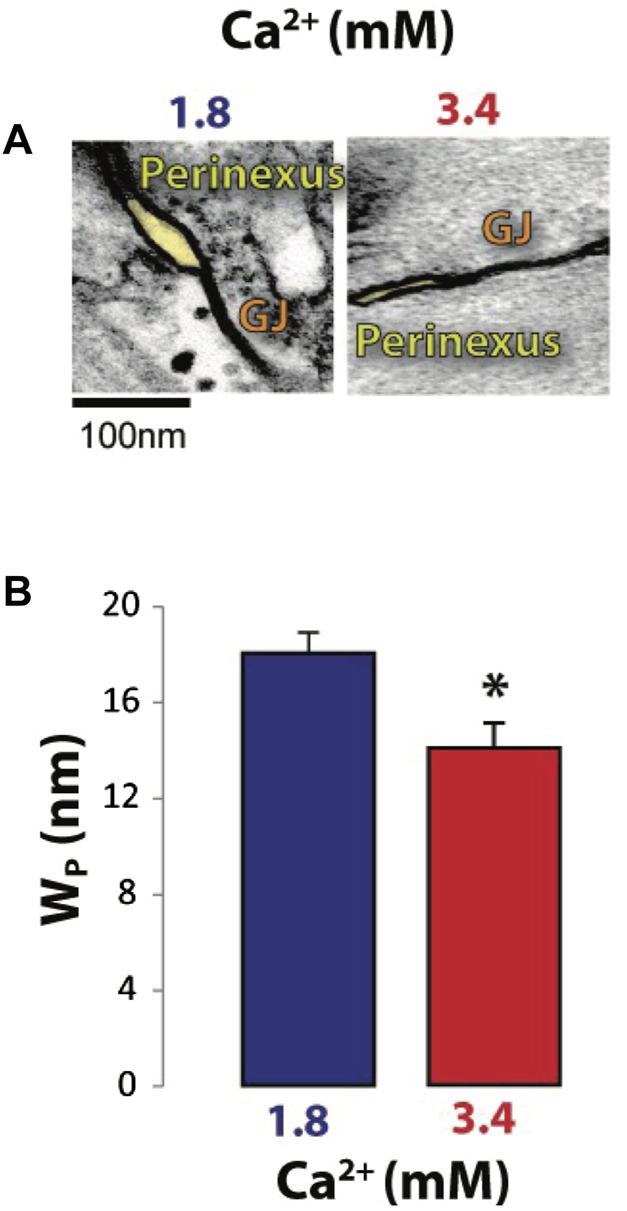
Increasing extracellular [Ca^2+^] decreases perinexal width (W_P_). **(A)** Representative TEM micrographs showing perinexal cleft sizes (shaded yellow). **(B)** Average perinexal width (W_P_); 3.4 mM extracellular [Ca^2+^] significantly reduced W_P_ relative to 1.8 mM extracellular [Ca^2+^]. *p < 0.05 with 2-tailed paired *t*-test, unequal variance*.*

Perfused heart studies were completed with WT and Cx43 HZ mice with 50% reduced Cx43 functional expression. Cx43 HZ mice provided a condition of low GJ coupling while the perfusate solution containing high calcium concentration (Table 1) was used to narrow W_p_ (Figure 5). (George et al., 2015; George et al., 2016) To confirm high [Ca^2+^] induced W_p_ narrowing, perinexal width was measured with TEM (Figure 5). Representative images of perinexal clefts during perfusion with typical extracellular [Ca^2+^] (1.8 mM) and high extracellular [Ca^2+^] (3.4 mM) are shown in Figure 5A. Higher extracellular [Ca^2+^] significantly reduced W_P_ (Figure 5B). This result is consistent with previous work (George et al., 2015; George et al., 2016).

**FIGURE 6:**
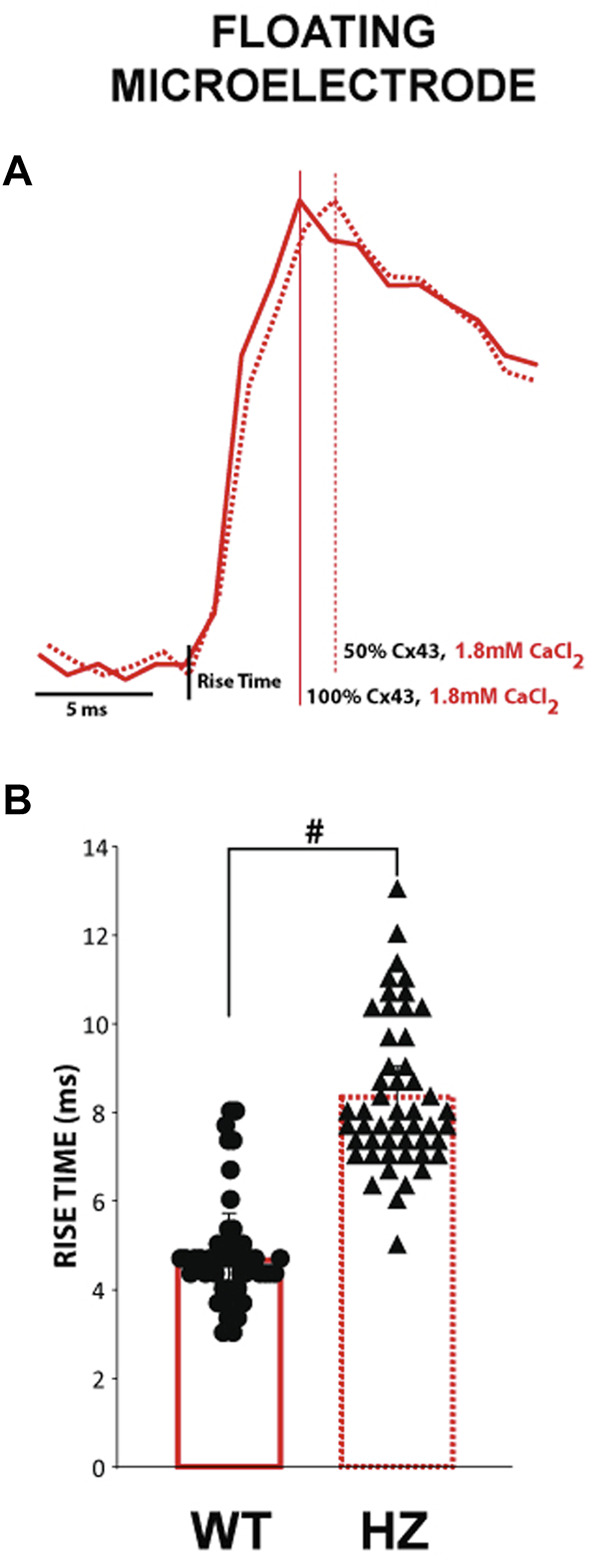
The time to peak for APs measured from Cx43 HZ hearts is greater than that of WT hearts. **(A)** Representative floating microelectrode recordings of the depolarization phase of ventricular APs measured along the transverse axis of propagation from an epicardially placed stimulating electrode. **(B)**. Summary data of the time from phase 4 to the peak of phase 0 depolarization (time to peak) from WT and Cx43 HZ hearts. Reducing Cx43 by 50% increased the time to peak. #, *p* < 0.01 with nested one-way ANOVA, (*n* = 3 WT hearts, *n* = 3 HZ hearts, 50 action potentials per heart).

The effect of reduced GJ coupling on action potential upstroke *rise time*, which is inversely proportional to *dV/dt_max,* was determined by comparing the depolarization phase of APs measured using floating glass microelectrodes in 3 WT and 3 Cx43 HZ mouse hearts. Representative phase zero transmembrane potentials for normal GJ coupling (WT -solid line) and reduced GJ coupling (Cx43 HZ - dashed line) are shown in Figure 6A. Although the curves are modestly different, average *rise time* is significantly longer for Cx43 HZ hearts compared to WT hearts (Figure 6B). This observation agrees with the simulation results.

**FIGURE 7:**
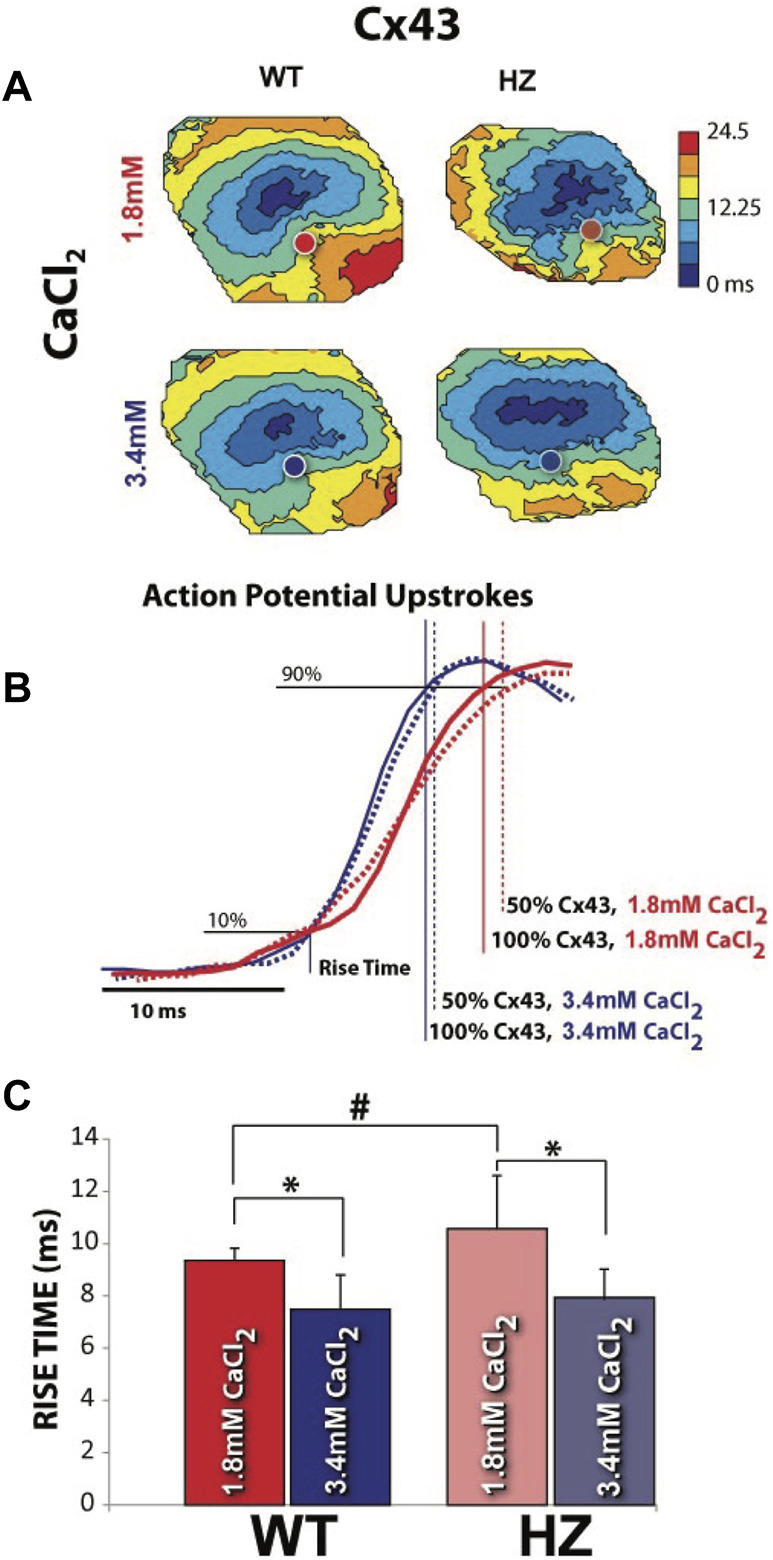
Increasing extracellular [Ca^2+^] to reduce perinexal width (W_P_) increased CV and decreased AP rise time. **(A)** Representative isochronal maps of activation times, with circles indicating where the APs shown in **(B)** were recorded. Closely spaced isochrones indicate slow conduction. **(B)** Measurement of rise time during the depolarization phase of optically mapped APs. Smaller W_P_ during perfusion with high extracellular [Ca^2+^] resulted in shorter rise times regardless of GJ coupling. Signals have been time-aligned at 10% of the total upstroke. **(C)** Average rise times for all WT and Cx43 HZ hearts perfused with normal and high levels of extracellular [Ca^2+^]. *p < 0.05 with 2 tailed paired *t*-test, unequal variance. #*p* < 0.05 with 2 tailed unpaired *t*-test. (*n* = 4, WT, 1.8 mM CaCl_2_, *n* = 10 WT 3.4 mM CaCl_2_, *n* = 5 HZ, 1.8 mM CaCl_2_, *n* = 7 HZ, 3.4 mM CaCl_2_).

Optical APs were mapped from the epicardium of 13 WT and 12 Cx43 HZ mouse hearts. The depolarization (phase zero) of APs along the transverse axis of propagation initiated by an epicardial stimulating electrode was analyzed to measure optical AP rise times. Data from hearts perfused with high [Ca^2+^] with narrower W_P_ were compared to data from hearts perfused with nominal [Ca^2+^] (Figure 7). Representative isochronal maps of activation times for all four combinations of GJ coupling and W_P_ are shown in (Figure 7A). Cx43 HZ hearts perfused with high [Ca^2+^] with narrower W_p_ exhibited increased CV, as noted by increased spread between isochrones compared to perfusion with normal [Ca^2+^] (Figure 7A). Representative optical APs during phase zero for each combination of GJ coupling and W_P_ are shown in Figure 7B. The observed slopes during phase zero demonstrate that the AP upstroke was much more sensitive to a reduction in W_p_ than a reduction in GJ coupling. This finding is also in agreement with the simulation results. In summary data, average *rise time* was longer for Cx43 HZ hearts than that of WT hearts when perfused with normal [Ca^2+^] (Figure 7C), consistent with the microelectrode results reported above and the simulation results. High [Ca^2+^], which corresponds to smaller W_p_, resulted in shorter rise times for HZ and WT hearts (Figure 6C) and therefore higher *dV/dt_max*. This effect of reduced W_P_ was maintained for WT and Cx43 HZ mouse hearts. Each of these observations are also in direct agreement with the simulation results.

## Discussion

AP propagation along a single fiber of myocytes having uniform or nonuniform ion channel distributions with variations in W_P_ and GJ coupling was simulated. The results support the following conclusions: 1) Distributing sodium and potassium ion channels predominantly to the ends of myocytes allowed for CV to be maintained when GJ coupling was low with nominal cleft width. 2) Phase zero AP depolarization along the edges of myocytes was primarily driven by depolarization at the ends of the myocytes. 3) Reduced W_P_ increased maximum *dV/dt* at the ends of myocytes for nonuniform ionic channel distribution. 4) A reduction in GJ coupling decreased maximum *dV/dt* primarily at the ends of cardiomyocytes in the non-uniform case when clefts were wide. 5) Phase zero, rise time was more sensitive to changes in W_P_ than to changes in GJ coupling.

Other numerical models of varying complexity that incorporated the 3D geometry of the intracellular and extracellular space have also shown that EpC plays an important role in CV (Kucera et al., 2002; Stinstra et al., 2005; Mori et al., 2007; Mori et al., 2008; Roberts et al., 2008; Ivanovic and Kucera, 2021; Moise et al., 2021). While our choice of numerical model may dictate the simulated CV values, the important features we have elucidated here will match other detailed models in the trends. The advantage of using our model of intermediate complexity is that it retains important geometric details, such as nonuniform ionic channels and GJ coupling, while providing computational efficiency. Likewise, a different ionic model, perhaps of greater detail or complexity, may provide different values but should provide similar trends in membrane depolarization rates. While we anticipate transverse conduction to show similar results in the investigation of ephaptic coupling and intracellular charge accumulation, such studies are left to future work.

In tandem with simulations, experiments were conducted with WT and Cx43 HZ mouse hearts perfused with solutions that induced similar physiological changes in W_P_. APs in the transverse direction were analyzed and conclusions 3, 4, & 5 above were confirmed. When GJ coupling was reduced, we found that conduction was preserved primarily by the dynamics in the narrow junctional clefts, which has a high density of sodium channels. As the cellular depolarization was slower with lower GJ coupling but faster with smaller W_P_, we did not observe an increased rate of sodium entry into the intracellular space that would be consistent with increased charge accumulation. We found that under conditions that arise during ischemia, ephaptic effects may play the most important role for potential therapies targeting arrhythmias caused by reduced conduction reserve.

Slowed conduction and conduction failure are important determinants of cardiac arrhythmias and sudden cardiac death. As the relationship between slowed conduction and risk for sudden death became more established, questions emerged regarding how the cardiac ultrastructure and underlying cellular structure determine the risk for conduction failure. Underlying anisotropic conduction raised the question of whether conduction was more likely to fail longitudinally or transversely to fibers (Tsuboi et al., 1985; Delmar et al., 1987; Balke et al., 1988; Turgeon et al., 1992). This proved to be a fairly complex area of study, with groups reporting the different conditions under which one axis of propagation is more likely to fail. With the understanding that GJs are principal determinants of conduction, significant efforts went into investigating the whole-heart electrophysiologic manifestations of GJ-mediated conduction. Spach et al. found that transverse conduction is slower, but that *dV/dt_max* is higher in the transverse relative to the longitudinal directions of propagation (Spach et al., 1981).

The fact that CV and *dV/dt_max* are relatively straight-forward to measure in a variety of tissue preparations (cell-cultures, papillary muscles, tissue slices, and whole-heart preparations), numerous studies sought to explore the relationship between these two parameters. Some studies in the 1980s performed in cardiac preparations revealed that the GJ inhibitor carbenoxolone decreased CV and *dV/dt_max* (Delmar et al., 1987), but this was mainly attributed to the finding that n-alkanols like heptanol and octanol reduce peak I_Na_ at concentrations within 1–2 mM (Oxford and Swenson, 1979; Nelson and Makielski, 1991). A year later, this understanding was revised when it was found that perfusion with octonal transiently increased *dV/dt_max* before decreasing it, with the conclusion that GJ uncoupling can increase *dV/dt_max* even as CV decreases. Again, about a year later in 1989, the relationship was explored with the volatile anesthetics halothane and enflurane, and the authors concluded that CV slowing and reduced *dV/dt_max* with these compounds occurred by a mechanism different from fast sodium channel inhibition (Ozaki et al., 1989). At approximately the same time, halothane was found to increase *dV/dt_max* in the transverse direction while decreasing it to a lesser extent in the longitudinal direction (Jalife et al., 1989). Over the ensuing years, investigators concluded that pharmacologic uncouplers can increase (carbenoxolone) (Dhillon et al., 2013), increase and decrease according to propagation direction (heptanol) (Jalife et al., 1989), not change (carbenoxolone) (Degroot et al., 2003) or decrease *dV/dt_max* [halothane and eloflurane (Delmar et al., 1987; Ozaki et al., 1989), palmatoleaic acid and octonol (Rohr et al., 1998), carbenoxolone (Entz et al., 2016; O'Shea et al., 2019)]. A more recent study suggested that at least one of the GJ uncouplers, carbenoxolone, can decrease perinexal width (Entz et al., 2016), which could explain at least one study that found carbenoxolone can increase *dV/dt_max* (Dhillon et al., 2013). However, significant additional experiments with GJ targeting compounds would be required to differentiate all the aforementioned studies while considering cell size, GJ functional expression, pharmacologic agent, and time course.

Despite the long history, clinical relevance, and exploration of pharmacologic agents’ mechanisms of action, drugs are sometimes not considered ideal experimental interventions given their concentration and use dependence, as well as off target effects. The CV-*dV/dt_max* relationship was then explored in transgenic mice with genetically reduced Cx43 functional expression, and those studies also provided mixed results. For example, genetic loss of Cx43 was associated with increased [neonatal Cx43± cultured strands (Thomas et al., 2003), and neonatal Cx43−/− hearts (Vaidya et al., 2001), adult Cx43± hearts (Eloff et al., 2001)] or no change (neonatal Cx43± cultures (Guerrero et al., 1997) isolated neonatal Cx43± cells (Fontes et al., 2012) and 2–4 month old conditional Cx43 KO (Mahoney et al., 2016)) in *dV/dt_max* or the optical correlate of rise time.

To our knowledge, the present work is the first to report that the optical correlate of rise time in the transverse direction of propagation from epicardial stimulation increases in 10–30 weeks of age in Cx43 HZ null mouse hearts relative to WT. Considered together with the many other pharmacologic studies, it is unsurprising that we found *dV/dt_max* is not predictably related to presumed GJ coupling, and the result may be an effect of perfusate composition, as we previously suggested for GJ coupling and CV (George and Poelzing, 2015). Furthermore, just as pharmacologic agents are associated with off-target effects, genetic manipulations, and specifically Cx43 for this discussion, are also associated with off target remodeling of other proteins important to conduction like Nav1.5 (Jansen et al., 2012; Agullo-Pascual et al., 2014). The literature also suggests that the observed relationship between GJ coupling and *dV/dt_max* may also be dependent on the age of myocytes and/or whether *dV/dt_max* was measured in isolated myocytes or in intact tissue.

Concurrent with experimental studies, computational models suggested that maximal inward current into a cell can increase with GJ uncoupling as CV decreases (Spach et al., 1981), which lead to subsequent models demonstrating that charge accumulation can first increase *dV/dt_max*, and then with further uncoupling will decrease *dV/dt_max* as down-stream sodium channels are insufficiently activated (Rudy and Quan, 1987; Shaw and Rudy, 1997b). Given the modest *dV/dt_max* change predicted for even a 50% reduction in GJ coupling, additional studies revealed that the relationship between *dV/dt_max* and CV is dependent on peak I_Na_ and cell size. Perhaps unsurprisingly, the present study now reveals that sodium channel localization plays an important role in the modulation of *dV/dt_max*. Coupled with previous computational and experimental studies demonstrating that CV is also complexly related to sodium channel localization and cell-to-cell separation within sodium channel rich intercalated disc clefts like the perinexus (George et al., 2015; Veeraraghavan et al., 2015; Entz et al., 2016; George et al., 2016; Veeraraghavan et al., 2016; Veeraraghavan et al., 2018; George et al., 2019; Hoeker et al., 2020; Nowak et al., 2020; King et al., 2021), this study supports previous work concluding that *dV/dt_max* is not a correlate of GJ coupling alone. Along those same lines, our finding that ephaptic mechanisms are also a determinant of *dV/dt_max* may go some way towards understanding the evidence for and against *dV/dt_max* as a correlate of peak I_Na_ in excitable cells (Weidmann, 1955; Hunter et al., 1975; Cohen and Strichartz, 1977; Hondeghem, 1978; Strichartz and Cohen, 1978; Walton and Fozzard, 1979; Cohen et al., 1981; Clarkson et al., 1984; Cohen et al., 1984; Roberge and Drouhard, 1987; Yamaoka, 1987; Sheets et al., 1988).

Given the complex mechanistic underpinnings of *dV/dt_max*, the experimental value of quantifying this parameter for understanding biophysical processes that underlie cardiac conduction and failure may be limited. However, the continued investigation of this parameter begins to reveal where conduction can fail, and that knowledge offers a more targeted approach to develop therapeutic strategies to prevent conduction failure, reentry, and arrhythmias. For example, *dV/dt_max* is often substantially lower in the lateral portions of the myocyte during enhanced EpC, suggesting that GJ coupling modulating EpC may differentially affect voltage-gated sodium channel kinetics along the lateral membranes and t-tubules of cardiomyocytes. Lastly, this study has important implications not only for ischemia, but also for sodium channel loss-of-function associated with hyperkalemia (King et al., 2021) and certain congenital forms of the Brugada Syndrome (Tsumoto et al., 2020).

## Data Availability

The raw data supporting the conclusion of this article will be made available by the authors, without undue reservation.
